# Emergency Department Presentation and Outcome of Children With Cyanotic Congenital Heart Diseases

**DOI:** 10.7759/cureus.17960

**Published:** 2021-09-14

**Authors:** Saadia Ilyas, Yasir Rehman, Ijaz Hussain, Assadullah Khan, Tausif Ahmed, Ali Akbar

**Affiliations:** 1 Paediatric Cardiology, Lady Reading Hospital, Medical Teaching Institute, Peshawar, PAK

**Keywords:** tetralogy of fallot, acyanotic congenital heart disease, cyanotic congenital heart disease, outcome, presentation

## Abstract

Objective

This study aimed to assess the outcome and see the spectrum of presenting symptoms of children with cyanotic congenital heart diseases (CHDs) admitted through the emergency department in Lady Reading Hospital (LRH) in Peshawar, Pakistan.

Materials and methods

This cross-sectional study included 104 children with cyanotic CHD admitted through the emergency department in the pediatric cardiology unit of LRH from February 2019 to January 2020. Diagnosis of cyanotic CHD was confirmed through two-dimensional echocardiography. Patients were treated according to standard protocols for their presenting symptoms. The data were analyzed using Statistical Package for the Social Sciences (SPSS), version 20.0 (IBM Corp., Armonk, NY) and frequencies were expressed as percentages.

Results

The study included 65 male cases (62.5%) and 39 female cases (37.5%), and participant ages ranged from 10 days to 15 years (mean, 2.7 ± 3.4 years). Of 104 patients, 70 presented with cyanotic spells (84.5%), 53 with fever (10.7%), 28 with respiratory distress (26.5%), 11 with loose stools (10.5%), 10 with hemiplegia (9.6%), and eight with seizures (7.6%). Pneumonia was a precipitating factor in 13 patients (12.5%), infective endocarditis in 12 patients (11%), gastroenteritis in 11 (11%), brain abscess and cerebrovascular accidents in seven patients each (6% each), meningitis in six patients (6%), and tuberculous pericardial effusion in one patient (<1%). The primary CHD was tetralogy of Fallot (TOF) in 52 patients, tricuspid atresia in 14 patients, pulmonary atresia in 13 patients, double outlet right ventricle in 10 patients, transposition of great arteries (TGA) in seven patients, and total anomalous pulmonary venous return (TAPVR), truncus arteriosus, congenitally corrected TGA, and critical pulmonary stenosis in two patients each. Twenty-six patients received treatment of the acute problem and instructions for follow-up monitoring. Twelve patients died during their hospital stay, and three left against medical advice. Fifteen patients received patent ductus arteriosus stenting, and six received right ventricular outflow tract stenting. One patient received a Blalock-Taussig (BT) shunt stent, and one received a left pulmonary artery stent. Two patients received a balloon pulmonary valvotomy, and one received pericardial effusion drainage. Eleven patients received surgical correction of TOF, 11 received surgical correction for BT shunt, four received surgical correction for brain abscess drainage, and two received TAPVR repair.

Conclusion

TOF was the most common cyanotic heart disease in our study. Cyanotic spells or increased cyanosis was the most common presenting concern. Cyanotic CHDs present with variable extracardiac signs and symptoms in emergencies. Appropriate assessment, immediate management of the acute problem, and timely intervention or surgical management result in good outcomes.

## Introduction

Cyanotic congenital heart disease (CHD) is a condition caused by compromised pulmonary blood supply, intracardiac mixing of oxygenated and deoxygenated blood, or both. The birth prevalence of major congenital heart defects is approximately 1% [1-3], and the estimated number of babies born worldwide with major heart defects is nine per 1,000 per year, with severe heart defects appearing in 2.5 per 1,000 births per year [4]. Although the incidence of cyanotic CHD is <25% of all cardiac defects [5], management and definitive treatment may be challenging. Patients with CHD present to the emergency department with various clinical symptoms that can affect multiple organ systems. Due to the complex management of this condition, clinicians must be aware of the common manifestations of cyanotic CHD to allow for prompt and accurate management [6].

This study aimed to evaluate cyanotic CHD's clinical presentation in children and assess their outcomes in the pediatric cardiology unit at the Lady Reading Hospital, Medical Teaching Institute (LRH-MTI) in Peshawar, Pakistan.

## Materials and methods

We conducted this cross-sectional study at the pediatric cardiology unit of LRH in Peshawar, Pakistan, from February 2019 to January 2020. The study included all pediatric cases of cyanotic CHD admitted through the emergency department. Diagnosis of cyanotic CHD was confirmed on echocardiography, performed by a pediatric cardiologist. Detailed histories were obtained from each patient, and all patients received an examination. Cyanotic CHDs were classified as tetralogy of Fallot (TOF) and its variants: tricuspid atresia, pulmonary atresia, double outlet right ventricle, transposition of great arteries (TGA), total anomalous pulmonary venous return (TAPVR), truncus arteriosus, congenitally corrected TGA, and critical pulmonary stenosis. We collected patient gender, age, presenting concerns, underlying cyanotic CHD, and outcomes. We used Statistical Package for the Social Sciences (SPSS), version 20.0 (IBM Corp., Armonk, NY) to analyze the data, and we calculated frequencies and means by descriptive analysis.

## Results

In twelve months, a total of 104 pediatric patients with cyanotic CHD were admitted through the emergency department, of whom 65 patients were male and 39 patients were female (age range: 10 days to 15 years; mean age: 2.7 ± 3.4 years). Table 1 lists the presenting concerns among study participants. The most common presenting concern was increased cyanosis or cyanotic spell in 70 patients (63.3%), followed by fever in 53 patients (50.9%). Pneumonia was the most common extracardiac factor in study participants (n=13; 12.5%) followed by infective endocarditis (n=12;11%). Table 2 presents all extracardiac conditions in children with cyanotic CHD presenting to the emergency department.

**Table 1:** Presenting concerns of children with cyanotic congenital heart disease

Presenting Concerns	N (%)
Spells/Increased Cyanosis	70 (67.3%)
Fever	53 (50.9%)
Respiratory Distress	28 (26.9%)
Loose stools	11 (10.5%)
Hemiplegia	10 (9.6%)
Seizures	8 (7.6%)

**Table 2:** Extracardiac conditions identified in children with cyanotic congenital heart disease presenting to the ED ED, emergency department; IE, infective endocarditis; PE, pericardial effusion.

Condition	N (%)
Pneumonia	13 (12.5%)
IE	12 (1.1%)
Gastroenteritis	11 (10%)
Brain Abscess	7 (6.7%)
Cerebrovascular Accident	7 (6.7%)
Meningitis	6 (5%)
Tuberculous PE	1 (0.9%)

The most common lesion was TOF and its variants in 52 patients (50%), followed by tricuspid atresia in 14 patients (13.4%). All cyanotic CHD lesions and their outcomes are presented in Table 3.

**Table 3:** Cyanotic congenital heart diseases and outcomes DORV, double-outlet right ventricle; TGA, transposition of great arteries; TAPVR, total anomalous pulmonary venous return; CCTGA, congenitally corrected transposition of great arteries.

Lesion	N (%)	Outcome (n)	
		Survived	Died
Tetralogy of Fallot	52 (50%)	48	4
Tricuspid atresia	14 (13.4%)	11	3
Pulmonary atresia	13 (12.5%)	12	1
DORV	10 (9.6%)	9	1
TGA	7 (6.7%)	7	0
TAPVR	2 (1.9%)	2	0
Truncus arteriosus	2 (1.9%)	0	2
CCTGA	2 (1.9%)	1	1
Critical pulmonary stenosis	2 (1.9%)	2	0

Sixty five out of hundred and four patients underwent surgical and catheter-based interventions and twenty-six patients received medical treatment for their acute condition (e.g., anemia, infection, and dehydration) and discharged with instructions for follow-up monitoring. Twelve patients died during their hospital stay, and three left against medical advice. Table 4 presents the interventions and surgical referrals for 65 patients, with patent ductus arteriosus stenting as the most common (n=15) followed by right ventricular outflow tract stenting (n=6). The most common surgical referrals were for Blalock-Taussig (BT) shunt (n=11) and total correction of TOF (n=11).

**Table 4:** Interventions and surgical referrals PDA, patent ductus arteriosus; BT, Blalock-Taussig; TOF, tetralogy of Fallot; TAPVR, total anomalous pulmonary venous return.

Intervention/Referral	N
PDA stenting	15
BT shunt	11
Total correction of TOF	11
Right ventricular outflow tract stenting	6
Atrial septostomy	4
Arterial switch	4
Brain abscess drainage	4
Pre-Glenn catheterization	3
Balloon pulmonary valvotomy	2
TAPVR repair	2
BT shunt stenting	1
Left pulmonary artery stenting	1
Pericardial effusion drainage	1

## Discussion

Given that relatively few studies have documented the presentation of cyanotic CHD in the emergency department, this study aimed to address this gap in the literature. The male predominance in our study population is also seen in other studies in Pakistan [7,8]; however, the male to female ratio for overall CHDs is 1:1 but varies in different types of CHDs [9].

Our most common type of cyanotic CHD was TOF, accounting for 50% of the conditions in our study population. This predominance of TOF was also noted in other local and international studies [7,10,11]. This might be because a relatively large number of genetic loci have been associated with TOF.

Increasing cyanosis was the most common presenting symptom followed by fever and respiratory distress, which aligns with findings reported by Al-Hamash [12] and Pradhan et al. [13]. Respiratory distress and fever are common presenting symptoms in studies that include cyanotic and acyanotic CHDs [14]. Pneumonia followed by infective endocarditis and gastroenteritis were the common systemic diseases presenting in our population of children with cyanotic CHD, while pneumonia followed by gastroenteritis and sepsis were reported by Masood et al. [14].

Catheter-based interventions to maintain pulmonary blood supply in cyanotic CHD patients showed promising results [15]. In our study, 12 patients died during their hospital stay and three left against medical advice. Most of these deaths could have been prevented if a primary repair was performed in time. Poverty, lack of education, and lack of specialized hospital units are likely the contributing factors in the morbidity and mortality of CHD patients. All the BT shunts and brain abscess drainage procedures in our population were performed in the same hospital, and all patients survived. Most of the total correction for TOF and total anomalous pulmonary venous return patients were referred outside the province due to a lack of specialized units.

Our study had some significant limitations. This was a hospital-based study and did not depict the actual prevalence of cyanotic CHD in the community. Community-based studies are required to determine the true incidence of this condition in pediatric patients. Further studies from multiple centers and follow-up studies are required in patients who have undergone intervention or surgical repair to expand the knowledge about the presentation and outcome of cyanotic CHDs.

## Conclusions

In our study, TOF was the most common cyanotic heart disease, accounting for half the emergency department presentations. Cyanotic spells or increased cyanosis was the most common presenting concern. Cyanotic CHDs present with variable extracardiac signs and symptoms in emergencies. Appropriate assessment, immediate management of the acute problem, and timely intervention or surgical management result in good outcomes. 
